# Digital Students’ Satisfaction With and Intention to Use Online Teaching Modes, Role of Big Five Personality Traits

**DOI:** 10.3389/fpsyg.2022.956281

**Published:** 2022-07-22

**Authors:** Sohaib Mustafa, Yu Qiao, Xin Yan, Aliya Anwar, Tengyue Hao, Sehrish Rana

**Affiliations:** ^1^College of Economics and Management, Beijing University of Technology, Beijing, China; ^2^Faculty of Education, University of Malaya, Kuala Lumpur, Malaysia; ^3^School of Economics and Management, North China Electric Power University, Beijing, China; ^4^Asia-Europe Institute, University of Malaya, Kuala Lumpur, Malaysia; ^5^Government Islamia Graduate College for Women, Faisalabad, Pakistan

**Keywords:** E-learning, digital teaching modes, personality traits, digital students, adoption intention, educational psychology, gender difference, SEM-ANN

## Abstract

During the COVID-19 pandemic, online teaching modes were found vital to continue students’ learning process, but sustainable implementation of online teaching models is an area of concern for policymakers. Psychiatrists are also eager to know students’ behavior toward learning and modes of teaching during COVID-19. We have drawn a model based on the big five personality traits to study students’ satisfaction with online teaching modes and their adoption intentions toward online teaching modes. We have collected data from 718 bachelor’s and master’s level students from four different universities. We have applied the SEM-ANN dual-stage approach to test personality traits’ influence and ranked them based on their normalized importance. The results revealed that agreeableness, conscientiousness, neuroticism, and openness positively influence students’ satisfaction with online teaching models, but that extraversion negatively influences their satisfaction. Agreeableness, extraversion, and neuroticism positively impact, but openness negatively influences. Conscientiousness does not affect adoption intention. Furthermore, agreeableness is the most significant, and conscientiousness is the least important factor for students to adopt online teaching modes. The findings of the study have useful perceptiveness for educational policymakers, academics, and psychiatrists.

## Introduction

Online learning through digital modes of teaching is prevailing since the revalorization of the IT sector and frequent use of the Internet. During COVID-19, students around the globe were offered to learn from home through online classes. It is mainly to control the spread of COVID-19 and keep the smooth learning process of students so their studies do not get disrupted.

Students who take up full-time teaching courses and enrolled in different universities generally have never encountered such kind of online classroom environment. They were not used to the mode of online teaching. It has some interesting outcomes, and students behave differently toward adopting the new teaching model. Students of courses that need physical encounters in laboratory works (medical, biomedical sciences, civil, mechanical, electrical, chemical, and material engineering) were less satisfied than those with no laboratory works (education, social sciences and humanities, accounting, and economics) (Eckhaus and Davidovitch, 2021). Furthermore, it has been observed that majority of students do not like online or E-teaching alone. This is most likely the case because of lack of social engagement. According to a recent survey in India, eighty percent of students believe that face-to-face learning is essential for practical learning, and seventy-seven percent of students believe that a classroom is a place for learning that is superior to other types of learning environments (Radha et al., 2020). The future of medical education lies in hybrid or blended learning, since it is more student-friendly and efficient (Hameed et al., 2020).

Researchers believe that satisfaction from a teaching mode or inclination toward its adoption or rejection is related to individual personality traits (Varela et al., 2012; Eckhaus and Davidovitch, 2021). Personality traits play a significant role in human decision-making (Irfan and Ahmad, 2021). It is also believed that online teaching and learning in an online mode are significantly associated with adopters’ perceptions. Students perceive it differently. It also affects students learning capacity and outcome (Coenen et al., 2021; Mateus et al., 2021; Rafique et al., 2021).

Recent studies have studied personality traits in different circumstances related to learning and education, such as online learning in the management field (Varela et al., 2012), Satisfaction from Online Teaching (Eckhaus and Davidovitch, 2021); still, they ignore the personality traits and their role in students satisfaction from online teaching mode and its adoption. Researchers have also studied the video conference mode of teaching in Vietnam and collected data from instructors, and measured the acceptance and use intention of a video conference (Nguyen and Nguyen, 2021); the same study is carried out in Pakistan (Akram et al., 2021), but they ignore the role of personality traits. They did not involve students in their study to understand their satisfaction with and adoption intention to online teaching modes.

Other studies have studied personality traits not in the context of online learning platforms but to learn the secondary level of students’ grade performance (Meyer et al., 2019) or personality traits’ role in satisfaction with training and vocational education (Volodina et al., 2019).

In addition to this, most studies have applied multivariate models such as SEM to conclude their results (Volodina et al., 2019; Akram et al., 2021; Nguyen and Nguyen, 2021), but researchers have suggested implementing dual-stage SEM-ANN models in studies that involve human psychology (Mustafa et al., 2021), because they provide a better picture of phenomena. Furthermore, study results of the first step (SEM) show a negative relationship between satisfaction and adoption intention that motivates us to carry on an ANN analysis to dig deeper on data and find a possible justification behind the surprising results.

With this brief discussion on the recently available literature, we have found a research gap that none of the recent studies involved students in studying their psychological state of mind toward satisfaction with online teaching modes or measured the intention to adopt online teaching modes in the future. Furthermore, the role of personality traits as significant antecedents of human decision-making has been ignored in recent studies. Hence, we have proposed the following research questions to fill this research gap.

**RQ1:** Do students’ personality traits play a significant role in their satisfaction with the online teaching model and its adoption in the future?

**RQ2:** What are the most significant (importance wise) personality traits in students’ online teaching model adoption?

This study is one of the first to examine students’ personality traits that influence their satisfaction with online learning and their adoption intentions of the online learning model in the future. To answer the research questions based on the five-factor theory, we have proposed a model and empirically tested it using a two-stage hybrid model, SEM-ANN. Apart from this, we have also examined gender-specific paths to understand the gender difference in online teaching models adoption, and personality traits that influence male and female segments. We have selected China to test our proposed model and collected data from four universities in China. The study results based on SEM (RQ1) revealed that agreeableness, conscientiousness, neuroticism, and openness positively influence students’ satisfaction with online teaching models, and that extraversion negatively influences it. For the adoption intention of these modes in the future, agreeableness, extraversion, and neuroticism positively influence, but openness negatively influences. Conscientiousness does not affect adoption intention. In response to RQ2, a sensitivity analysis revealed that agreeableness is the most significant predictor according to the normalized importance of variables, followed by neuroticism and extraversion. The study findings have beneficial implications for researchers and psychiatrists to understand students’ psyche. They also yield practical implications for policymakers for students.

## Theoretical Framework

### Five-Factor Theory

The foundation of the five-factor model (McCrae and Costa, 1992) is laid on personality characteristics that serve as fundamental components of an individual’s character. The model comprises five personality traits: conscientiousness, neuroticism, agreeableness, extraversion, and openness to experience. Conscientiousness is linked to a nature that is more goal-oriented, organized, determined, perseverant, and dedicated, whereas neuroticism is related to feelings of insecurity, distress, guilt, anger, depression, anxiety, and aggression (Talwar et al., 2022). Neuroticism is a negative emotional state that leads to emotional instability (Talwar et al., 2022). Next, extraversion is linked to qualities like expressiveness, sociability, and outgoingness, while openness to experience is connected with qualities such as creativity, curiosity, imagination, and a broad-minded mindset. In conclusion, agreeableness is connected to characteristics such as compassion, caring, generosity, trust, and cooperativeness. In a further development of the paradigm (Olson and Evans, 1999), noted that people’s emotions are influenced by their levels of extraversion and neuroticism, while their levels of Agreeableness, Conscientiousness, and Openness to experience, impact their cognitive processes. Based on the five-factor theory, we have developed a model to understand students’ satisfaction from the online class model and their intentions to adopt this teaching model in the future. It will explain students psyche toward online delivery of lectures and learning through online modes of teaching during COVID-19. We have proposed the following conceptual framework to study the subject.

### Literature Review and Hypothesis Development

**FIGURE 1:**
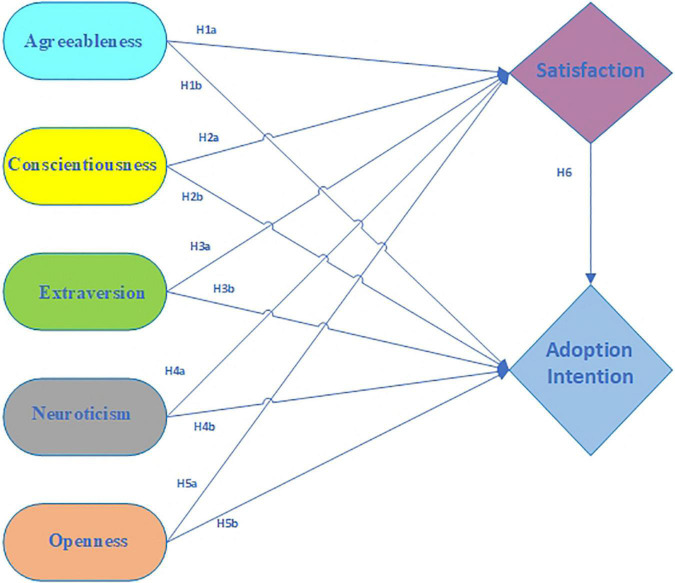
Conceptual framework.

The level of academic performance has been evaluated based on a variety of different variables (Gladius Jennifer et al., 2018; Mahmoodi et al., 2019; Belsare et al., 2020; Mateus et al., 2021), but the focus is typically placed on sociological ones such as the environment or the dynamics of the family, psycho-pedagogical ones such as study habits or attitudes toward the university, and purely psychological ones such as cognitive processes or personality. According to the findings, academic success is related to intellectual characteristics, but it is also an impact of a multitude of adaptative, behavioral, and psychopathological variables, including certain personality traits (Mateus et al., 2021). Recent studies have studied the influence of personality traits on preferences and educational choices (Coenen et al., 2021), personality traits and burnout (Narang et al., 2022), academic performance in higher education (Mateus et al., 2021), domain-specific achievement (Meyer et al., 2019), satisfaction with training and vocational education (Volodina et al., 2019), education and biosecurity compliance (Racicot et al., 2012), associations among gender, education, religious faith, and social class personality traits (Furnham and Cheng, 2015), early childhood pedagogies and views about education (Smidt et al., 2015), students’ use of YouTube for educational purposes during the course of COVID-19 (Elareshi et al., 2022), and so on. Still, none of the studies have investigated the effect of students’ personality traits on their satisfaction with and adoption of online teaching modes offered during COVID-19. To fill this research gap, we have proposed the conceptual model presented in Figure 1.

### Agreeableness

As a behavioral trait, “agreeableness” indicates a person’s prosocial attitude, commitment, and responsiveness (Irfan and Ahmad, 2021). When it comes to controlling emotions in interpersonal situations, it is especially concerned with “belonging,” a framework that encourages tight ties and provides social support (Talwar et al., 2022). Therefore, agreeableness is a reflection of individual variances in prosocial and communicative inclinations, which may range from warmth and attachment to animosity and antagonism (Shiner and Masten, 2008). People who have high agreeableness to disagreeability ratio are polite. They have a goal of maintaining uniformity even if doing so would be detrimental to their interests. An agreeable person is trustworthy, kind, and avoids arguments to foster a healthy learning environment. Agreeableness is related to interpersonal skills and characterizes someone who tends to be agreeable (Irfan and Ahmad, 2021). Thus, they behave like a group and are open to new technologies (Irfan and Ahmad, 2022). Recent studies stated that agreeableness is the best predictor of mathematics, science, engineering, and technology specialization and preferences (Coenen et al., 2021). In another study, a negative correlation was found between agreeableness and students’ grades across both genders (Meyer et al., 2019). Researchers have also figured out that agreeableness is positively correlated with vocational education and training satisfaction (Volodina et al., 2019). Another study in British found a significant correlation between agreeableness and both religious background and frequency of religious service attendance (Furnham and Cheng, 2015). Another study in Germany found that respondents who scored higher on the agreeableness scale had a greater chance of belonging to the ambitious profile than those who scored higher on the moderate scale (Smidt et al., 2015; Wang and Sohail, 2022). With this discussion, we have hypothesized that:

**H1a:** Students’ agreeableness personality trait will positively influence their satisfaction with online learning/teaching modes during COVID-19.

**H1b:** Students’ agreeableness personality traits will positively influence their adoption intention of online learning/teaching modes during COVID-19.

### Conscientiousness

A person’s propensity to be responsible, well-organized, and self-disciplined is what is meant to be understood when talking about their conscientiousness. People who demonstrate the attribute of conscientiousness are more likely to participate in administrative activities. They will not give up on pursuing their objectives no matter how much effort or time is necessary (Irfan and Ahmad, 2021). These people are more likely to gather accurate and relevant knowledge and have a greater propensity to work hard to achieve their goals (Irfan and Ahmad, 2022). Those who scored higher on both the conscientiousness and openness scales had a greater likelihood of being categorized as ambitious than those who are reserved (Smidt et al., 2015). Another study in British revealed a significant correlation between conscientiousness and both religious background and frequency of religious service attendance (Furnham and Cheng, 2015). Researchers have also claimed that conscientiousness positively influences training and vocational education satisfaction (Volodina et al., 2019). A study on upper secondary education in Germany revealed no correlation between conscientiousness and test results in English regardless of the other factors included. Conscientiousness was shown to have substantially favorable benefits in mathematics even after controlling for gender, socioeconomic status, and cognitive ability (Meyer et al., 2019). Although it is generally accepted that conscientiousness is the most important of the big five in terms of school achievement, there is very little correlation between conscientiousness and desire for the science, engineering, technology, or mathematics field as a particular area of concentration (Coenen et al., 2021). With a detailed discussion of recent studies’ results, we proposed the following:

**H2a:** The conscientiousness trait of students’ personality will positively influence their satisfaction from online teaching/learning modes during COVID-19.

**H2b:** The conscientiousness trait of students’ personality will positively influence their adoption of online teaching/learning modes during COVID-19.

### Extraversion

Extroverts are often self-assured and like being in the company of others. These traits tend to go hand in hand. People that have a high level of extraversion tend to be outgoing, self-assured, and like new experiences (Irfan and Ahmad, 2022). Coenen et al. (2021) claimed that personality characteristics are somewhat less relevant in connection to the actual specialty choice, for which they uncover a strong (and sizeable) negative link with extraversion. They further claim that the best indicator of a person’s true decision in the science, engineering, technology, and mathematics fields is extraversion. A study conducted in Germany by researchers with the aim of “Educational Outcomes of Students from Vocational and Academic Upper Secondary Schools” claims that in all models, extraversion was shown to have a detrimental impact on final mathematics examinations and test scores. None of the models found any statistically significant impacts on English (Meyer et al., 2019).

Furthermore, researchers figured out that the extraversion trait is positively associated with training and vocational education satisfaction (Volodina et al., 2019). The extraversion trait is also found positively significant in religious background and frequency of religious service attendance (Furnham and Cheng, 2015). A study conducted in Germany with the title of “Relations between the Big Five personality traits of prospective early childhood pedagogues and their beliefs about the education of preschool children” found an insignificant relationship of the extraversion trait (Smidt et al., 2015). With this literature, we proposed that:

**H3a:** The extraversion trait of students’ personality will negatively influence their satisfaction from online teaching/learning modes during COVID-19.

**H3b:** The extraversion trait of students’ personality will positively influence their adoption intention of online teaching/learning modes during COVID-19.

### Neuroticism

The term “neuroticism” refers to distressing emotions including sadness, agony, frustration, dread, and unease (Nagel et al., 2018). People who suffer from neurosis are more sensitive to effects of external stimuli and have a propensity to overreact to ordinary events. They become more anxious as a consequence of these stimuli, which leads to decline in cognitive activities and increased vulnerability to stress (Irfan and Ahmad, 2022). A study revealed that people who exhibited neuroticism had a greater likelihood of being part of the ambitious profile with strong endorsements of educational ideas (Smidt et al., 2015; Yen et al., 2018). Neuroticism showed a negative relationship with religious background, frequency of religious service attendance (Furnham and Cheng, 2015), and training and vocational education satisfaction (Volodina et al., 2019), but had educational choices and preferences in the science, engineering, technology, and mathematics fields (Coenen et al., 2021). However, researchers measuring “domain-specific achievement in upper secondary education” in Germany claimed that English grade and overall grade point average demonstrated favorable associations with neuroticism. This might be connected to the more meticulous learning behavior connected with neuroticism, which is advantageous for cumulative evaluation but not for direct testing scenarios. On the other hand, direct testing settings can be beneficial for cumulative assessment (Meyer et al., 2019). We also suggest that negative emotions may lead to a negative outcome from the students’ perspective, and we hypothesize that:

**H4a:** The neuroticism trait of students’ personality will negatively influence their satisfaction from online teaching/learning modes during COVID-19.

**H4b:** The neuroticism trait of students’ personality will negatively influence their adoption intention of online teaching/learning modes during COVID-19.

### Openness

Studies on people’s personalities have shown that openness is a good indicator of whether or not they are likely to be inventive, progressive, or adventurous (Stajkovic et al., 2018). They are naturally inquisitive and eager to acquire new knowledge, which enables them to provide fresh ideas and points of view. Openness to experience and agreeableness with others have been shown to be the greatest indicators of preferences in technical fields (Coenen et al., 2021). A study conducted at a Colombian university did not find any clue of a significant relationship between openness and academic performance in higher education (Sohail and Delin, 2013; Mateus et al., 2021). Another study found a strong positive correlation between openness to experience and satisfaction with training and vocational education; however, there was no correlation between openness to experience and overall life satisfaction (Volodina et al., 2019). A British cohort was studied to investigate associations between openness qualities, religious beliefs, and service attendance (Furnham and Cheng, 2015), and researchers found a negative association between openness with outcomes. With discussion of the literature, we suggest that openness to experience will lead to higher satisfaction as students explore new means of learning that can lead to their learning satisfaction during COVID-19. Keeping in mind the circumstances of COVID-19, the adoption intention may not be favorable because students who took classes that required physical encounters in the form of laboratory works (such as medical, biomedical sciences, civil, mechanical, electrical, chemical, and material engineering) reported lower levels of satisfaction than students who took classes that did not require laboratory works (such as education, social sciences and humanities, accounting, and economics) (Eckhaus and Davidovitch, 2021). Hence, we hypothesize that:

**H5a:** The openness to experience trait of students’ personality will positively influence their satisfaction from online teaching/learning modes during COVID-19.

**H5b:** The openness to experience trait of students’ personality will negatively influence their adoption intention of online teaching/learning modes during COVID-19.

### Satisfaction and Adoption Intention

Satisfaction from technology leads to adoption and use of technology or product. Researchers have found that satisfaction from 5G services leads to their adoption in the future (Mustafa et al., 2022d), electronic gadgets (Mustafa et al., 2022a), electronic vehicle adoption (Mustafa et al., 2021), the effect of climate change and adoption techniques to minimize risk (Sohail et al., 2022b), green transportation adoption (Sohail et al., 2021). Furthermore, researchers have revealed that the level of satisfaction experienced by users will have a favorable and substantial influence on their desire to continue using academic library applications (Rafique et al., 2021). Satisfaction from training and vocational education leads to life satisfaction (Volodina et al., 2019), but students that engage in degrees/courses that require practical work may not like to use them in the future because of their learning requirements (Eckhaus and Davidovitch, 2021). Hence, we suggest that satisfaction from online learning modes may or may not influence students to use them in the future. Hence we propose:

**H6:** Satisfaction from online teaching/learning platforms during the COVID-19 period will influence their adoption intention in future.

### Gender Influence

We have decided to study gender-specific paths to clarify the role of personality traits in online teaching/learning mode’s satisfaction and adoption. Male and females differently perceive things and have different psych toward adopting a technology (Mustafa et al., 2022a) and contributing knowledge to online Q and A communities (Mustafa and Wen, 2022; Mustafa et al., 2022c). The same is found in the case of personality traits; gender differences in personality traits and the influence of personality traits are different for men and women (Narang et al., 2022). A significant gender difference is found in a study conducted at a Colombian university to measure academic performance in higher education (Mateus et al., 2021). Hence, we suggest that there might be a different influence of personality traits in our study.

## Methodology

### Research Context

To test our proposed model and check the influence of the big five personality traits on satisfaction and adoption intention of online learning modes, we picked four universities in China. It was estimated that there would be 2,738 public colleges and universities in China in the year 2020. There were 1,270 universities and 1,468 higher vocational colleges included in this total (Statista, 2022). In China’s public universities and colleges in the year 2020, there were around 33 million students enrolled in undergraduate degree programs. It was estimated that 18.3 million of them were enrolled in bachelor’s degree programs, and that the remaining 14.6 million were enrolled in short-cycle degree programs that were more realistically oriented (Statista, 2021; Li et al., 2022). Hence, these incredible statistics encourage us to study personality traits that influence E-learning adoption and student satisfaction from these modes in China.

### Construction of Instrument and Sample Collection

We employed a construct that has been shown useful in past studies. Supplementary Material fully describes each measuring item’s configuration and characteristics. We make very few adjustments to the ideas for our research constructs so that they may better serve the objective of our investigation. We discussed the instrument with three academics who are specialists in the field, and we included all of their insights. After that, we conducted a pilot study to determine respondents’ time answering a questionnaire and their comments. Thirty graduate students (master’s and bachelor’s) were selected to participate in the pilot study. Throughout the pilot study, we talked with academic specialists to include any new content necessary to clearly understand student’s behavior. In the next stage, we collaborate with two translators fluent in Chinese. Language specialists adapt the English version of the instrument for use in the Chinese language. After that, we retranslate the instrument into English to determine whether or not the newly translated version properly calculates the same answer as the instrument’s initial version. Again, academic specialists were engaged to ensure that the translated version of the instrument included the same importance in both English and Chinese when it came to the construct items. In the end, fifteen Chinese students at the master’s level were selected to participate in the final instrument validity. They gave encouraging feedback, and the results of the second phase of the pilot project supported the continuation of the investigation; nevertheless, participants of the pilot study were not included in the final sample.

Because of the potential for manual data input and other types of human error, we decided to conduct the poll online. We used a five-point Likert scale to collect the cross-sectional dataset and measure the response of our target population, with 1 representing “strongly disagree” and 5 “strongly agree.” Researchers claim that a five-point Likert scale is easy to manage and less complex than higher-order scales (Awan et al., 2021; Gul et al., 2021). We have randomly picked the Students of Bachler and master’s level enrolled in Beijing University of Technology, Xiangtan University, Wuhan University, and Shanghai Normal University. A total of eight hundred questionnaires *via* the Chinese star questionnaire^1^ QR code was shared with students of the economics, computer science, mathematics, and education fields (Two hundred from each university). We received 718 useable samples with a response rate of 89.75%. The sample size of 718 is large enough to carry out further statistical analysis as it has more than 10 responses per item threshold (Yen et al., 2017; Sohail et al., 2022a; Zhongjun et al., 2022).

### Demographic of Respondents

We collected basic demographic information about the respondents. Table 1 represents their demographic characteristics.

**TABLE 1:** Demographic characteristics of the sample.

Characteristics	Range	Frequency	Percentage
Gender	Male	388	54%
	Female	330	46%
Age	18–22	360	50.2%
	22–24	358	49.8%
Degree Level	Bachelor	360	50.2%
	Master	358	49.8%
Enrolled Major	Economics	213	29.6%
	Computer science	179	24.9%
	Mathematics	192	26.7%
	Education	134	18.7%
Enrolled University	Beijing University of Technology	190	26.4%
	Xiangtan University	189	26.3%
	Wuhan University	168	23.3%
	Shanghai Normal University	171	23.8%

### Common Method Bias

Common method bias (CMB) might result when the same response technique is used to measure the dependent and independent variables. A study’s validity might be jeopardized by common methodological biases. Previous researchers recommend several procedural and statistical measures to avoid CMB (Podsakoff et al., 2012). We informed all the participants about the study’s purpose and use of data. To every respondent, the questionnaire was explained in detail before they responded to it. There were also language experts and academics during the preparation process of the questionnaire to make it clear and easy to understand for the respondents. Before handing over a questionnaire QR code, we explained to the respondents that there were no specific criteria for picking the correct answer. The respondents need to provide their own perceptions and response. We made clear that it will not be treated as wrong or right. In the analysis part, we performed the recommended Harman’s single-factor analysis, and the results revealed that the single factor contains only 37.91% of the total variance, which is far less than the recommended threshold value of 50% (Podsakoff et al., 2012; Mustafa et al., 2022e). Hence, there is no serious threat of CMB existing in our study.

### Partial Least Squares Structural Equation Modeling

Partial least squares structural equation modeling was the method that we used since it is the one that is suggested most for usage in situations a the study is focused on predicting and examining dependent variables in order to explain the highest amount of variation. According to Roldán and Sánchez-Franco (2012), the PLS-SEM methodology is the most successful prediction-oriented strategy. In addition to this, it can concurrently process measurement (outer) and structural (inner) models. In addition to this, it is suitable for analysis of intricate route models (Hair et al., 2016). Last but not least, PLS-SEM can work with a very small sample size while producing more accurate findings. As a result, it seems that PLS-SEM is the method that should be used for this investigation. As was recently pointed out by Mustafa et al. (2022d), the PLS-SEM approach is on the rise, which may be attributed to the potential advantages it offers in management science (Mustafa et al., 2021, 2022d). According to Hair et al. (2020), the PLS path modeling approach is evaluated in two stages in order to guarantee that the constructs’ measurements are correct and reliable: (a) the evaluation of the measurement model determines the outer mode’s reliability and validity and (b) the assessment of the structural model defines the inner model or the link among the latent constructs. However, because of the fact that the model incorporates non-linear interactions, it is beneficial to carry out an analysis that consists of two stages.

### Multivariate Assumptions

Researchers claim that multivariate assumptions need to test, i.e., linearity, multicollinearity, and homoscedasticity, before performing a multivariate analysis (Mustafa et al., 2021, 2022d). A Kolmogorov-Smirnov (K-S) test was performed with intention of determining whether or not the data follow a normal distribution; however, the findings show that the data do not follow a normal distribution (Hew et al., 2017). Supplementary Material provides evidence for both linear and non-linear interactions that take place between dependent and independent variables. Last but not least, VIF scores were examined in order to determine whether or not the model had any collinearity issues. According to the findings of this research, all of the variables have VIF values that are less than 5 (Table 2). According to Hair et al. (2016), VIF values lower than 5 indicate no collinearity problems associated with a dataset. The indicators’ loadings and cross-loadings can be found in the Supplementary Material.

**TABLE 2:** Reliability and validity analysis.

Variables	Items	Loadings	T-statistics	VIF	α	CR	AVE
Agreeableness	AGR1	0.636***	16.775	1.852	0.843	0.885	0.565
	AGR2	0.678***	20.524	1.933			
	AGR3	0.747***	25.97	1.911			
	AGR4	0.820***	32.094	2.615			
	AGR5	0.802***	30.107	2.302			
	AGR6	0.808***	29.332	2.370			
Adoption Intention	AI1	0.874***	39.26	2.068	0.842	0.905	0.76
	AI2	0.887***	52.55	2.226			
	AI3	0.855***	47.545	1.824			
Conscientiousness	CONS1	0.843***	21.158	2.215	0.889	0.923	0.75
	CONS2	0.890***	28.357	2.496			
	CONS3	0.864***	23.528	2.367			
	CONS4	0.866***	24.215	2.443			
Extraversion	EXTR1	0.820***	14.745	1.724	0.848	0.898	0.689
	EXTR2	0.698***	11.593	1.478			
	EXTR3	0.896***	25.063	2.945			
	EXTR4	0.890***	20.374	2.996			
Neuroticism	NEUR1	0.817***	24.05	2.326	0.91	0.928	0.65
	NEUR2	0.844***	28.565	2.698			
	NEUR3	0.872***	29.1	3.225			
	NEUR4	0.797***	22.26	2.419			
	NEUR5	0.769***	21.811	1.922			
	NEUR6	0.729***	16.7	1.990			
	NEUR7	0.807***	20.858	2.064			
Openness	OPEN1	0.848***	23.858	2.365	0.86	0.899	0.641
	OPEN2	0.806***	16.845	2.061			
	OPEN3	0.795***	18.717	1.841			
	OPEN4	0.761***	17.298	1.708			
	OPEN5	0.790***	16.536	1.905			
Satisfaction	SAT1	0.863***	26.364	1.643	0.757	0.859	0.672
	SAT2	0.870***	23.644	1.786			
	SAT3	0.716***	14.791	1.366			

***Significant at p < 0.001.

### Measurement Model

According to what was found in the study (Hair et al., 2016; Jamil et al., 2022), the reliability of the indicator, construct convergent validity, and discriminant validity should be used to evaluate measurement models. Cronbach’s Alpha (α) and indication loading was used in the process of determining the instrument’s level of reliability. Convergent validity testing was performed on the indicators of the constructs to see whether or not they provide an accurate assessment of the research variables. AVE is an abbreviation for the total amount of variation in indicators that was compensated for by the latent construct, and CR is an abbreviation that describes the consistency of the variables. The reliabilities of the individual items are presented in Table 2, and their reliability is evaluated based on the factor loadings of the items on the respective constructs. For a component to be deemed significant and be included in the model, its factor loading must be equal to or higher than 0.6 (Hair et al., 2016; Jamil et al., 2021). In addition, the significance of Cronbach’s Alpha for all constructs is higher than or very near to the acceptable threshold of 0.7 (Werts et al., 1974).

Additionally, the constructs’ composite reliability (CR) was evaluated because it is generally accepted that CR is a more effective tool for measuring reliability than Cronbach’s alpha (Werts et al., 1974). The fact that the CR values of constructs are all higher than 0.7 is an additional factor that strengthens the reliability of the variables. Concurrently, the estimates for the convergent validity of the average variance extracted (AVE) were all higher than 0.5, proving that convergent validity had been achieved (Table 2; Hair et al., 2016).

Finally, the Fornell–Larcker criterion is used so that the discriminant validity of the model may be evaluated. Table 3 shows that discriminant validity has been established in accordance with the Fornell–Larcker criterion. This is shown by the fact that the top value of the association of measures is greatest in each column (Fornell and Larcker, 1981; Hair et al., 2016).

**TABLE 3:** Discriminant validity with the Fornell–Larcker criterion.

	Mean	Std	AGR	AI	CONS	EXTR	NEUR	OPEN	SAT
AGR	4.05	0.74	0.792						
AI	4.08	0.86	0.756	0.872					
CONS	3.7	1.08	0.425	0.415	0.866				
EXTR	3.7	1.04	0.443	0.511	0.66	0.83			
NEUR	3.89	0.81	0.565	0.571	0.42	0.338	0.806		
OPEN	3.95	0.85	0.53	0.395	0.569	0.284	0.548	0.801	
SAT	3.79	1.03	0.612	0.439	0.451	0.286	0.531	0.572	0.819

*AGR, agreeableness; AI, adoption intention; CONS, conscientiousness; EXTR, extraversion; NEUR, neuroticism; OPEN, openness; SAT, satisfaction; STD, standard deviation.*

### Structural Model Assessment

Assessment of the structural model is the second step in the PLS-SEM evaluation process. In order to evaluate a structural path model, it is necessary to evaluate the multicollinearity of data and the predictive relevance of the model, empirical significance of path coefficients, and degree of confidence (Hair et al., 2016). In the current research, general guidelines were used for the purpose of analyzing the structural model and interpreting the data (Hair et al., 2016).

**FIGURE 2:**
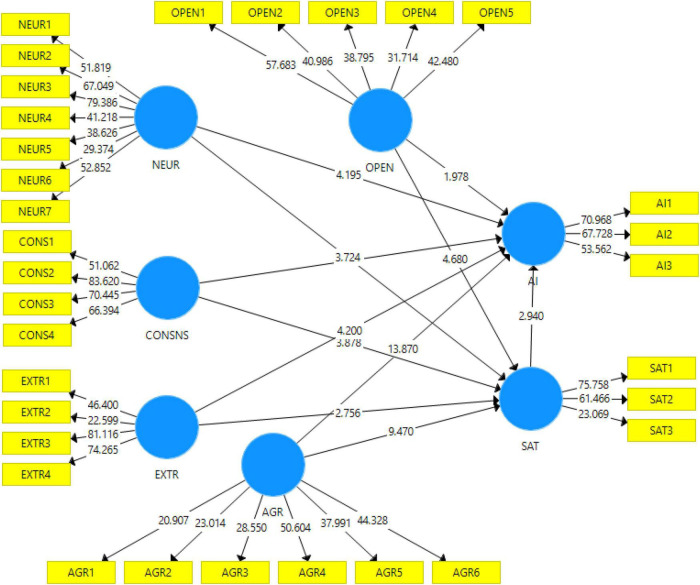
Path model [partial least squares structural equation modeling (PLS-SEM)].

In our scenario, there are two different endogenous constructs (Figure 2). Hence the PLS-SEM path analysis results revealed that for satisfaction, the *R*^2^ value is 0.489 (*Q*^2^ = 0.321), and for adoption intention, *R*^2^ is.697 (*Q*^2^ = 0.522). Our model explains the 48.9% variance caused by the independent variables in satisfaction and 69.7% in AI (Table 4). Predictive relevance is measured by Q^2^, which indicates whether or not a model has predictive relevance. In our model, the *Q*^2^ values indicate that the predictive relevance of the endogenous constructs is established.

**TABLE 4:** Path analysis [partial least squares structural equation modeling (PLS-SEM)].

	Beta	Standard deviation	T-statistics
AGR → AI	0.711***	0.05	14.129
AGR → SAT	0.383***	0.04	9.499
CONS → AI	0.006*^NS^*	0.05	0.116
CONS → SAT	0.173***	0.045	3.86
EXTR → AI	0.177***	0.042	4.225
EXTR → SAT	–0.114***	0.04	2.835
NEUR → AI	–0.218***	0.052	4.174
NEUR → SAT	–0.163***	0.044	3.721
OPEN → AI	–0.09**	0.044	2.04
OPEN → SAT	0.215***	0.045	4.718
SAT → AI	–0.114***	0.037	3.043
	DV = SAT		
*R* ^2^	0.489		
Adjusted *R*^2^	0.485		
*Q* ^2^	0.321		
	DV = AI		
*R* ^2^	0.697		
Adjusted *R*^2^	0.694		
*Q* ^2^	0.522		

***Significant at p < 0.001, **significant at p < 0.05.

*NS, not supported; DV, dependent variable; AGR, agreeableness; AI, adoption intention; CONS, conscientiousness; EXTR, extraversion; NEUR, neuroticism; OPEN, openness; SAT, satisfaction; STD, standard deviation.*

In order to evaluate the established hypotheses, we first examined the direct correlations between the variables and then conducted a bootstrapping test with 5,000 replicates following Mustafa et al. (2021) and Mustafa et al. (2022d). The results revealed the AGR (β = 0.711; *p* < 0.001) for AI and (β = 0.383; *p* < 0.001) for SAT, CON (β = 0.006; *p* = 0.908) for AI and (β = 0.173; *p* < 0.001) SAT, EXTR (β = 0.0.177; *p* < 0.001) for AI and (β = –0.114; *p* = 0.005) for SAT, NEUR (β = 0.218; *p* < 0.001) for AI and (β = 0.163; *p* < 0.001) SAT, OPEN (β = –0.09; *p* = 0.041) for AI and (β = 0.215; *p* < 0.001) for SAT, and SAT (β = –0.114; *p* = 0.002) for AI. These confirmed the proposed hypotheses H1a, H1b, H2b, H3a, H3b, H4a, H4b, H5a, H5b, and H6, and only H2a is rejected (Table 4).

Furthermore, to access the group-specific effects for both genders, we conducted PLS-MGA and reported the bootstrapped results for men and women individually. We used conducted 5000 resampling in bootstrapping to ensure the architecture in this model is the same as that in model 1.

The results presented in Table 5 for both genders reveal that CONS has no influence on AI, and that EXTR has no influence on SAT (for both genders). At the same time, NEUR does not influence male students’ satisfaction, and OPEN influences male students’ adoption intention. The rest of the relationships was found to be significant for both genders.

**TABLE 5:** Bootstarping results for PLS-MGA.

Paths	Female sample			Male sample		
	Beta	STDEV	*T*-value	Beta	STDEV	*T*-value
AGR → AI	0.706***	0.063	11.272	0.71***	0.08	8.85
AGR → SAT	0.374***	0.053	7.119	0.425***	0.063	6.717
CONS → AI	–0.085*^NS^*	0.068	1.244	0.105*^NS^*	0.062	1.707
CONS- > SAT	0.17***	0.057	2.986	0.159**	0.073	2.167
EXTR → AI	0.206***	0.058	3.558	0.215***	0.052	4.112
EXTR → SAT	–0.085*^NS^*	0.052	1.641	–0.114*^NS^*	0.077	1.474
NEUR → AI	0.358***	0.08	4.499	0.118**	0.059	2.02
NEUR → SAT	0.241***	0.062	3.861	0.103*^NS^*	0.075	1.368
OPEN → AI	–0.14**	0.06	2.333	–0.109*^NS^*	0.056	1.925
OPEN → SAT	0.167***	0.069	2.422	0.231***	0.066	3.495
SAT → AI	–0.161**	0.057	2.841	–0.1**	0.047	2.137

***Significant at p < 0.001, **significant at p < 0.05.

*NS, not supported; DV, dependent variable; AGR, agreeableness; AI, adoption intention; CONS, conscientiousness; EXTR, extraversion; NEUR, neuroticism; OPEN, openness; SAT, satisfaction; STD, standard deviation.*

### Artificial Neural Network

An ANN is a non-linear statistical data simulation tool that can be trained several times to enhance model performance (Mustafa et al., 2022b). An ANN is used for prediction and categorization of data. ANN models are better than other multivariate models in prediction, but they are not suitable for testing hypotheses because of the presence of “BLACBOX” (Mustafa et al., 2021; Talwar et al., 2022). Researcher claim that an ANN can be combined with SEM to achieve better and more reliable results, especially if multivariate assumptions are violated or the sample size is small because an ANN does not need any multivariate assumption and works perfectly with a low dataset. As observed (Supplementary Material), the dataset has some non-linear interaction, so it is better for two-stage analysis. In addition to this, in response to RQ2, we want to rank the predictors according to their normalized importance by sensitivity analysis for students’ E-learning mode adoption intention; hence, we used an ANN in the second stage of the analysis.

**FIGURE 3:**
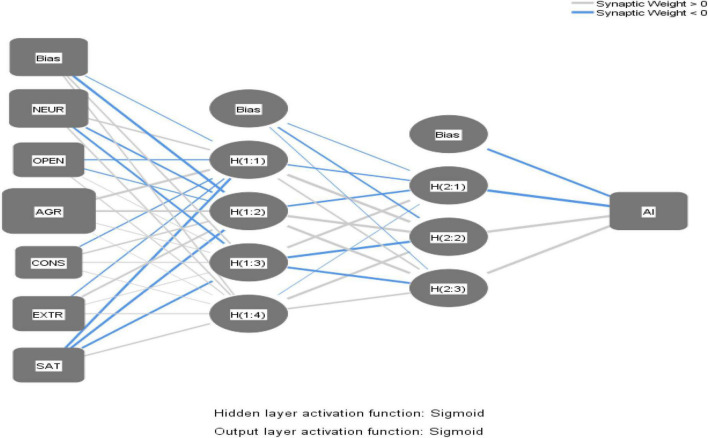
Artificial neural network (ANN) model for adoption intention (AI).

We constructed an ANN model for AI, and we picked the sigmoid as the activation function and two hidden layers for neurons following early researchers (Mustafa et al., 2021; Figure 3). In order to prevent over-fitting the model, a method called 10-fold cross-validation was used, and 90 percent of the data were used for training while only 10 percent was used for testing (Table 6).

**TABLE 6:** Root mean square of errors (RMSE) values for training and testing.

Training			Testing			
*N*	SSE	RMSE	*N*	SSE	RMSE	Total sample
638	4.539	0.084	80	0.479	0.077	718
634	4.78	0.087	84	0.659	0.089	718
641	4.83	0.087	77	0.611	0.089	718
648	6.369	0.099	70	0.321	0.068	718
645	4.468	0.083	73	0.259	0.060	718
645	6.782	0.103	73	0.875	0.109	718
640	4.711	0.086	78	0.471	0.078	718
639	4.219	0.081	79	0.634	0.090	718
645	4.364	0.082	73	0.596	0.090	718
634	4.742	0.086	84	0.458	0.074	718
Mean	4.980	0.088	Mean	0.536	0.082	
Std Dev	0.868	0.007	Std Dev	0.178	0.014	

*R^2^ = 1–RMSE/S^2^, where S^2^ is the variance of the test data’s desired output.*

*N, number of samples; RMSE, root mean square of errors.*

*AGR, agreeableness; CONS, conscientiousness; EXTR, extraversion; NEUR, neuroticism; OPEN, openness; SAT, satisfaction served as the input neurons.*

*AI, adoption intention served as the output neuron.*

Table 6 presents the root mean square of errors (RMSEs). An RMSE represents the predictive power of a model (Mustafa et al., 2022b; Talwar et al., 2022). In our model, the RMSE values (Table 6) for training (mean = 0.088, STD = 0.007) and testing (mean = 0.082, STD = 0.014) represent the high predictive power of the constructed ANN model for AI.

**FIGURE 4:**
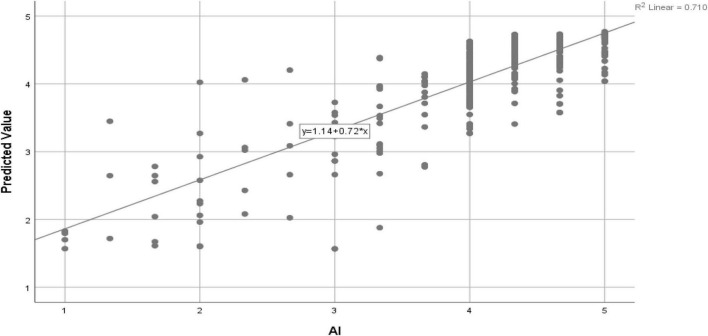
Regression standard residuals for artificial neural network (ANN) model.

In addition, to evaluate the efficiency of the ANN models, we determined a goodness-of-fit coefficient analogous to the *R*^2^ value found in the regression models based on a specific methodology (Figure 4).

### Sensitivity Analysis

A sensitivity analysis was carried out by making use of the ANN model, and the outcomes are presented in Table 7. The importance of the inputs was validated by the hidden layer of the neural model, which had synaptic weights that were not zero. The output of the model is very sensitive to changes in the values of the various inputs, which are then used to determine the “relative significance” of each factor. We were able to determine the normalized relevance of each variable with the assistance of these findings by conducting a ratio calculation with regard to the value that was found to be the highest overall. The findings of the sensitivity analysis are provided in Table 7.

**TABLE 7:** Sensitivity analysis [artificial neural network (ANN) model for adoption intention (AI)].

Neural network	NEUR	OPEN	AGR	CONS	EXTR	SAT
NN-1	0.526	0.274	1.000	0.138	0.303	0.285
NN-2	0.407	0.206	1.000	0.132	0.282	0.174
NN-3	0.456	0.173	1.000	0.047	0.263	0.210
NN-4	0.491	0.168	1.000	0.091	0.402	0.143
NN-5	0.534	0.263	1.000	0.174	0.297	0.325
NN-6	0.652	0.112	1.000	0.085	0.414	0.073
NN-7	0.372	0.203	1.000	0.136	0.186	0.262
NN-8	0.395	0.231	1.000	0.077	0.287	0.199
NN-9	0.418	0.237	1.000	0.135	0.209	0.259
NN-10	0.503	0.231	1.000	0.149	0.297	0.232
Average importance	0.475	0.210	1.000	0.116	0.294	0.216
Normalized importance	47.53%	20.99%	100%	11.64%	29.39%	21.61%

*AGR, agreeableness; AI, adoption intention; CONS, conscientiousness; EXTR, extraversion; NEUR, neuroticism; OPEN, openness; SAT, satisfaction; STD, standard deviation.*

Based on the sensitivity analysis results presented in Table 7, agreeableness, with normalized importance of 100%, is ranked first, followed by neuroticism (47.53%) and extraversion (29.39%), while conscientiousness (11.64%), openness (20.99%), and satisfaction (21.61%) are least important factors for student adoption intention.

## Discussion

This study aims to analyze the influence of personality traits (big five) on students’ satisfaction with online teaching modes and their adoption intention for educational policymakers and psychiatrists to understand the students’ psyche during COVID-19 and the influence of online teaching modes on their output. We have presented a model based on the big five personality traits. We have used an SEM-ANN hybrid model to test the proposed hypothesis (RQ1) and rank the variables according to their normalized importance (RQ2).

H1a–H6 measure the direct effect of understudy variables on students’ satisfaction with online teaching modes and their adoption intentions in the future. H1a presents the relationship between agreeableness and students’ satisfaction with online teaching models, while H1b presents the relationship between agreeableness and adoption intention. The study results revealed that agreeableness has a significant positive relationship with both. With this, we can conclude that students’ personality aspects of inclination, proactiveness, and attentiveness urge them to use online modes during COVID-19 and avoid possible loss of learning during the period. Students act responsibly and adopt new means of teaching to carry on in a healthy learning environment. It is consistent with earlier studies (Volodina et al., 2019; Coenen et al., 2021) but contradicts studies that claim it negatively impacts students’ grades (Meyer et al., 2019). H2a and H2b present the relationship between conscientiousness and students’ satisfaction with online learning modes and their adoption. The results revealed that although conscientiousness has a positive correlation with satisfaction, it does not significantly impact the adoption intention of online teaching modes. It reflects that although students are well-organized and self-disciplined during the COVID-19 period and comply with teaching policies, they are not willing to adopt this method of teaching in the future. It seems realistic, as many students who need a practical work to complete their learning cannot do it online. It is consistent with Volodina et al. (2019) and Coenen et al. (2021). H3a and H3b present the personality trait “extraversion,” which influences students’ satisfaction and adoption intention toward online learning modes. As extravert people like to be in groups and like new experiences, they prefer to study in groups or face to face as they interact with fellow students. This is the reason that the personality trait of being Extraversion negatively correlates with students’ satisfaction with online teaching modes as they have no physical interaction with fellow students, but in the meantime, we have found a positive relationship with adoption intention means Extraversion those like to have new experience and self-assured are more inclined to adopt online teaching mode in future. Possibly they want to find something new and exciting in the online teaching mode. It is consistent with Volodina et al. (2019) but contradicts Meyer et al. (2019). H4a and H4b describe the relationship between neuroticism and explained variables. The results revealed that neuroticism has a negative association with satisfaction with and adoption intention of online teaching modes. Neuroticism represents distressing emotions, and being in isolation during COVID-19 may negatively influence students’ psyche, and they are not satisfied with long-term online teaching without any physical interaction (face-to-face teaching/learning). It negatively influences their intentions to the adopt online mode of teaching. It is consistent with previous studies (Volodina et al., 2019; Coenen et al., 2021). H5a and H5b present the relationship of openness with students’ satisfaction with and adoption intention of online teaching modes. The results revealed that openness positively influences students’ satisfaction but negatively impacts their adoption intention. The possible reason behind the results can be that students who are open to experience are satisfied with online teaching. Still, the influence of neuroticism or other negative states is so high that they do not want to adopt these modes in the future. Finally, H6 presents the influence of satisfaction and adoption intention. Although in previous studies, if users are satisfied with a technology they intend to adopt it (Rafique et al., 2021; Mustafa et al., 2022d). We found surprising results in our study; although the students are satisfied with online teaching modes in given circumstances (COVID-19), they are not willing to adopt the methods of teaching in the future. A possible justification can be that the students want to resume face-to-face classes and physical interactions with peers, and laboratory works. The students believe that online teaching models may not be good in the long run and may result in low learning and less professional training. As indicated in a recent study, 80% of students think face-to-face learning is important for practical learning, and 77% of students prefer a classroom as a better environment for learning (Radha et al., 2020). This is also consistent with ideas presented by earlier researchers (Eckhaus and Davidovitch, 2021).

Furthermore, we conducted PLS-MGA and studied the gender-specific influence of personality traits in male and female students. The results revealed that there is a significant difference between male and female behaviors. Conscientiousness has no influence on adoption intention for both genders, and neither does the trait “extraversion” affect the satisfaction of both genders. We have observed that the effect of extraversion on satisfaction was significant and negative in the overall sample. In addition to this, the female students’ neuroticism positively influences their level of satisfaction for online teaching, but it does not influence the male students. Openness was negatively significant for female students’ adoption intention but did not affect male students’ adoption. We can say that there is a significant difference between male and female students’ behaviors toward satisfaction and adoption, as neuroticism and openness influence the female students but not the male students. It is consistent with the claim of researchers that gender plays a role in students’ performance (Mateus et al., 2021; Narang et al., 2022).

Finally, to answer RQ2, we conducted ANN modeling and a sensitivity analysis to identify the most influential factors behind students’ adoption intention. The sensitivity analysis revealed that agreeableness ranked first, followed by neuroticism and extraversion, and that conscientiousness, openness, and satisfaction are the least important factors for student adoption intention. The ANN results serve as the robustness of the SEM results and provide a clear picture and a deep analysis. We have observed that satisfaction is number four according to its importance for students, which strengthens the results of H6. Furthermore, conscientiousness is the least important among all the traits, which supports our findings of rejection of H2b.

## Theoretical Contributions and Suggestions

The study results provide substantial theoretical contributions to the available literature. This study is one of the first to analyze personality traits and their influence on students’ satisfaction with online teaching modes and their intentions to adopt online teaching methods in the future. We have provided new insights into the available literature by incorporating students’ big five personality traits in the context of online teaching modes during COVID-19. We have improved and given a new direction to earlier studies that used personality traits as predictors of human behavior in learning (Volodina et al., 2019; Coenen et al., 2021; Eckhaus and Davidovitch, 2021; Mateus et al., 2021; Nguyen and Nguyen, 2021; Rafique et al., 2021; Narang et al., 2022).

We have provided a new insight into educational psychiatrists that students’ psyche is different during COVID-19, and that they perceive and behave differently toward satisfaction and adoption of online teaching modes. Furthermore, we provided evidence that male and female students’ satisfaction with online teaching modes and their adoption intentions significantly vary with gender.

We have also applied the dual-stage hybrid model (SEM-ANN) and proved that it provides deep insight and robust results; hence, we recommend applying it in human psychology-related studies. Furthermore the ANN model explained the variance much better (71%) than SEM. In the light of the study findings, we suggest that policymakers, academics, and psychiatrists consider the ranking provided in our study to target students’ psyche during COVID-19 and attain better results from online teaching modes by engaging students.

The findings provide a new avenue for policymakers to rethink whether to continue online learning or shift it to a hybrid model if COVID-19 persists. We suggest that policymakers focus on a teaching model where students are involved in a practical work in person and deliver theoretical knowledge online.

### Limitations and Future Recommendation

Apart from contribution, the study has some limitations that can lead to new research directions. First, we collected our responses from a single country. Personality traits’ influence on students’ behaviors can fluctuate with different geographical boundaries and availability of infrastructure such as in developing countries where the internet is not frequently available. Students may face problems in attending online classes. Second, we treated the students from different academic backgrounds as one; there are possibilities that practical degree students, and students from non-practical degree enrollment have a different attitudes toward online teaching modes. Therefore, a thorough study based on academic background can provide much better and more specific policy recommendations to policymakers. Third, we measured the students’ satisfaction and their adoption intention of online teaching modes. Future studies can study how the personality traits can help in achieving maximum satisfaction and motivate students to adopt online teaching modes. Lastly, we suggest carrying out a study that measures the moderation effect of personality traits on students’ behavior.

## Conclusion

Based on the study findings, we have concluded that students are satisfied with online teaching modes under a given circumstance (COVID-19), because they do not want to face worse outcomes of not learning. However, they are not inclined to adopt online teaching methods in the future as a permanent teaching mode. We have also concluded that the big five personality traits do not unanimously influence students’ behaviors, but that they have different levels of influential strength. Furthermore, the big five personality traits influence males’ and females’ intentions and satisfaction differently.

## Data Availability Statement

The datasets presented in this study can be found in online repositories. The names of the repository/repositories and accession number(s) can be found below: https://drive.google.com/file/d/1FhjGY2aPywrgWBvnggBj5bmTP2JlapHw/view?usp=sharing.

## Author Contributions

SM: conceptualization, methodology, software, and writing—original draft. YQ and XY: review of the final draft, editing, visualization, handling of data flow, and managing data. SR, AA, and TH: investigation and data collection. All authors contributed to the article and approved the submitted version.

## Conflict of Interest

The authors declare that the research was conducted in the absence of any commercial or financial relationships that could be construed as a potential conflict of interest.

## Publisher’s Note

All claims expressed in this article are solely those of the authors and do not necessarily represent those of their affiliated organizations, or those of the publisher, the editors and the reviewers. Any product that may be evaluated in this article, or claim that may be made by its manufacturer, is not guaranteed or endorsed by the publisher.

## Footnotes

1
www.wjx.cn

## Abbreviations

RQ: research question
SEM: structural equation model
ANN: artificial neural network
PLS-SEM: partial least squares structural equation modeling
CMB: common method bias
VIF: variance inflation factor
α: Cronbach’s alpha
AVE: average variance extracted
AGR: agreeableness
AI: adoption intention
CONS: conscientiousness
EXTR: extraversion
NEUR: neuroticism
OPEN: openness
SAT: satisfaction
STD: standard deviation.
