# Supplementary material for: Digital Students’ Satisfaction With and Intention to Use Online Teaching Modes, Role of Big Five Personality Traits
Source: Front Psychol. 2022 Jul 22;13:956281. doi: 10.3389/fpsyg.2022.956281 (PMC9354135; doi:10.3389/fpsyg.2022.956281)
Supplement: Supplementary file 1 [file Table_1.DOCX]

**Table 1. Survey Questionnaire construct and items**

| **Construct** | **Items** | **Reference** |
| --- | --- | --- |
| **Openness (OPEN)** | **OPEN 1:** I am eager to experience online teaching mode. | (Irfan & Ahmad, 2021) |
|  | **OPEN 2:** The appealing mode of online teaching attracts my attention. |  |
|  | **OPEN 3:** Using an online teaching model seems exciting. |  |
|  | **OPEN 4:** I am insightful that online teaching modes can revolutionize teaching modes. |  |
|  | **OPEN 5:** Online teaching model is a source of experiencing new and useful technology. |  |
| **Conscientiousness (CONS)** | **CONS 1:** If I could afford it, I would use online teaching services immediately. | (Irfan & Ahmad, 2021) |
|  | **CONS 2:** I need to prioritize my plans if I need to use online teaching modes. |  |
|  | **CONS 3**: I can decide if I need to use online teaching modes. |  |
|  | **CONS 4:** I am free to make my decisions about learning models. |  |
| **Extroversion (EXTR)** | **EXTR 1:** I often talk to my friends/colleagues/relatives about online teaching models. | (Irfan & Ahmad, 2021) |
|  | **EXTR 2**: I can convince my friends to opt for online teaching modes. |  |
|  | **EXTR 3:** I often discuss in my social circle about online teaching modes. |  |
|  | **EXTR 4**: I feel confident about learning online modes. |  |
| **Agreeableness (AGRE)** | **AGRE 1:** I agree to help if my friends/colleagues/relatives are in need. | (Irfan & Ahmad, 2021) |
|  | **AGRE 2:** I agree to provide help to my needy friends if I am good at using online teaching modes. |  |
|  | **AGRE 3**: I agree to use online teaching modes if my university/teachers want me to do so. |  |
|  | **AGRE 4**: I generally share my network expertises with my needy friends. |  |
|  | **AGRE 5**: I agree to make a use decision if I like the service/mode. |  |
|  | **AGRE 6**: I always keep my promises and commitments. |  |
| **Neuroticism (NEUR)** | **NEUR 1**: The online teaching modes do not fascinate me. | (Irfan & Ahmad, 2021) |
|  | **NEUR 2**: Transferring to the online teaching model is a complex decision. |  |
|  | **NEUR 3:** Transferring to an online teaching model is time-taking and problematic. |  |
|  | **NEUR 4**: I am particularly not eager to change my learning modes. |  |
|  | **NEUR 5**: Using online teaching modes may negatively impact my learning capacity. |  |
|  | **NEUR 6:** If most of my friends do not opt for an online teaching model, I will not use either. |  |
|  | **NEUR 7:** Online teaching models are not an effective solution to face to face teaching models. |  |
| **Adoption intentions (AI)** | **AI 1:** I intend to continue using online teaching modes in the future. | (Mustafa, Zhang, Shehzad, Anwar, & Rubakula, 2022) |
|  | **AI 2:** I plan to continue to use Online teaching methods frequently. |  |
|  | **AI 3**: I will recommend online teaching/learning modes to others. |  |
| **Satisfaction (SAT)** | **SAT 1:** I am satisfied with the 5G performance. | (Mustafa et al., 2022) |
|  | **SAT 2**: I am fully satisfied with 5G services. |  |
|  | **SAT 3:** I feel satisfied by my experience of using 5G internet. |  |

**Table 2. Cross loadings.**

|  | **AGR** | **AI** | **CONS** | **EXTR** | **NEUR** | **OPEN** | **SAT** |
| --- | --- | --- | --- | --- | --- | --- | --- |
| **AGR1** | 0.636 | 0.414 | 0.383 | 0.267 | 0.523 | 0.464 | 0.658 |
| **AGR2** | 0.678 | 0.426 | 0.47 | 0.306 | 0.564 | 0.538 | 0.728 |
| **AGR3** | 0.747 | 0.543 | 0.216 | 0.304 | 0.315 | 0.344 | 0.339 |
| **AGR4** | 0.82 | 0.722 | 0.226 | 0.351 | 0.382 | 0.31 | 0.314 |
| **AGR5** | 0.802 | 0.731 | 0.297 | 0.393 | 0.349 | 0.354 | 0.345 |
| **AGR6** | 0.808 | 0.724 | 0.304 | 0.36 | 0.394 | 0.368 | 0.358 |
| **AI1** | 0.716 | 0.874 | 0.327 | 0.461 | 0.516 | 0.317 | 0.365 |
| **AI2** | 0.657 | 0.887 | 0.374 | 0.454 | 0.466 | 0.347 | 0.429 |
| **AI3** | 0.709 | 0.855 | 0.384 | 0.422 | 0.509 | 0.369 | 0.356 |
| **CONS1** | 0.33 | 0.329 | 0.843 | 0.615 | 0.366 | 0.461 | 0.333 |
| **CONS2** | 0.441 | 0.4 | 0.89 | 0.556 | 0.391 | 0.545 | 0.465 |
| **CONS3** | 0.347 | 0.364 | 0.864 | 0.537 | 0.384 | 0.486 | 0.379 |
| **CONS4** | 0.339 | 0.334 | 0.866 | 0.591 | 0.309 | 0.466 | 0.366 |
| **EXTR1** | 0.425 | 0.489 | 0.521 | 0.82 | 0.285 | 0.264 | 0.235 |
| **EXTR2** | 0.279 | 0.258 | 0.516 | 0.698 | 0.222 | 0.222 | 0.243 |
| **EXTR3** | 0.4 | 0.473 | 0.588 | 0.896 | 0.314 | 0.265 | 0.272 |
| **EXTR4** | 0.342 | 0.43 | 0.574 | 0.89 | 0.288 | 0.189 | 0.203 |
| **NEUR1** | 0.44 | 0.455 | 0.292 | 0.244 | 0.817 | 0.466 | 0.406 |
| **NEUR2** | 0.481 | 0.444 | 0.355 | 0.265 | 0.844 | 0.494 | 0.473 |
| **NEUR3** | 0.437 | 0.425 | 0.349 | 0.244 | 0.872 | 0.479 | 0.516 |
| **NEUR4** | 0.394 | 0.384 | 0.314 | 0.208 | 0.797 | 0.401 | 0.417 |
| **NEUR5** | 0.453 | 0.385 | 0.503 | 0.339 | 0.769 | 0.527 | 0.409 |
| **NEUR6** | 0.268 | 0.336 | 0.26 | 0.229 | 0.729 | 0.317 | 0.297 |
| **NEUR7** | 0.62 | 0.691 | 0.308 | 0.352 | 0.807 | 0.402 | 0.439 |
| **OPEN1** | 0.484 | 0.393 | 0.472 | 0.261 | 0.491 | 0.848 | 0.461 |
| **OPEN2** | 0.388 | 0.267 | 0.424 | 0.207 | 0.375 | 0.806 | 0.424 |
| **OPEN3** | 0.412 | 0.296 | 0.455 | 0.242 | 0.424 | 0.795 | 0.462 |
| **OPEN4** | 0.375 | 0.27 | 0.441 | 0.172 | 0.388 | 0.761 | 0.464 |
| **OPEN5** | 0.45 | 0.342 | 0.479 | 0.249 | 0.502 | 0.79 | 0.478 |
| **SAT1** | 0.6 | 0.395 | 0.395 | 0.264 | 0.494 | 0.552 | 0.863 |
| **SAT2** | 0.52 | 0.396 | 0.397 | 0.26 | 0.444 | 0.498 | 0.87 |
| **SAT3** | 0.344 | 0.271 | 0.308 | 0.162 | 0.351 | 0.321 | 0.716 |


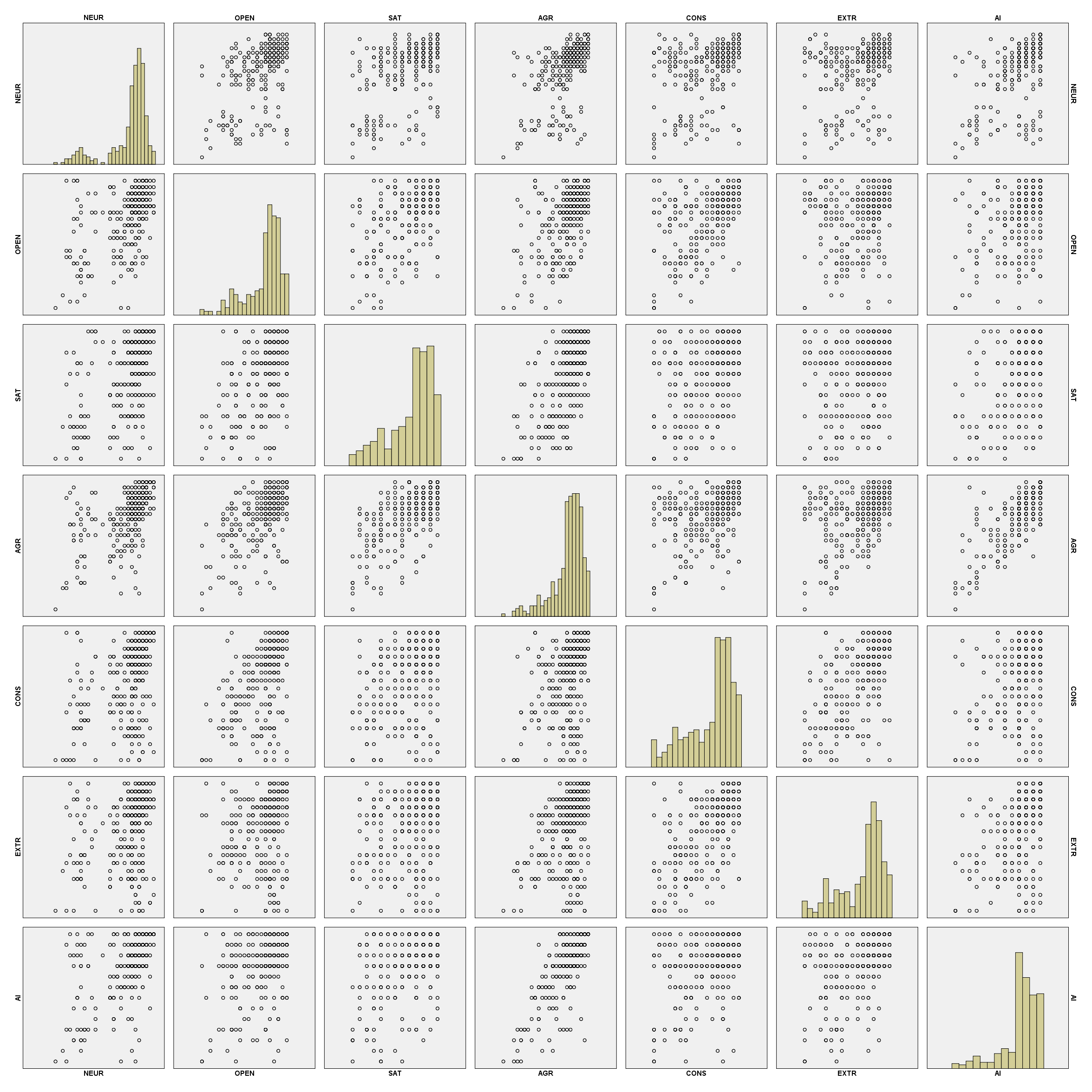


**Figure 1 . Data linearity**

**References:**

Irfan, M., & Ahmad, M. (2021). Relating consumers' information and willingness to buy electric vehicles: Does personality matter? *Transportation Research Part D: Transport and Environment, 100*, 103049. doi:<https://doi.org/10.1016/j.trd.2021.103049>

Mustafa, S., Zhang, W., Shehzad, M. U., Anwar, A., & Rubakula, G. (2022). Does Health Consciousness Matter to Adopt New Technology? An Integrated Model of UTAUT2 With SEM-fsQCA Approach. *Front Psychol, 13*. doi:10.3389/fpsyg.2022.836194
